# A Case of Triple Reimplantation

**Published:** 1899-05

**Authors:** Arthur Zentler

**Affiliations:** Paris, France


### Abstracts and Translations.

### A CASE OF TRIPLE REIMPLANTATION.

BY DR. ARTHUR ZENTLER, PARIS, FRANCE.

This is as a contribution to the study of a question that has been treated so frequently and with authority that I believe it will be interesting to cite this simple case of reimplantation.

It deals with a child of fourteen years of age, of good constitution, who was presented for our advice on the 21st of October, 1897, after an accident that had determined the loss of three teeth in the lower jaw,—namely, the incisors, two left and one right.

The patient showed us the teeth, which he had in his pocket.

It was two days after the accident that we saw the case, and, an interesting circumstance to note, the work of granulation had already commenced.

After having taken an impression of the jaw, to study the conditions with which we would have to contend, the following wash was ordered:

Chlorate of potassium, 8 grains;
Fluid extract of hamamelis, 125 grains.
One spoonful in a half-glass of hot water, as a lotion, every two hours.

We recommended the patient to return the following day.

The usual advice had been given him with regard to diet, preparatory to the anæsthesia and the ether. We then proceeded to the preparation of the teeth; resection of the extreme apex; extraction of pulp and filling of the canals with the aid of gutta-percha. The filling was completed by a resisting bed of cohesive gold, the whole slightly polished. . . .

The antiseptic precautions were scrupulously observed for preserving the teeth in a solution of bichloride of mercury 1 to 1000 after a previous cleansing with a soft brush and soap.

The next day, before proceeding with the operation, we made a careful wash of the mouth with peroxide of hydrogen; the seat of operation was particularly the object of our attention.

Ether was administered, and during the sleep a trepan well sterilized was introduced in the sockets to lift up all the new-forming tissue. The trepan was succeeded by a round instrument designed to enlarge the sockets mentioned. The teeth were tried in their respective sockets, then removed, and the sockets filled with cotton dipped in a solution of bichloride 1 to 1000.

When the patient regained consciousness, the cotton was removed, the sockets washed with hot water, and finally with hydrogen dioxide, the teeth were put in place, joined, and tied with threads of silk.

The patient was discharged with a recommendation to wash the mouth every hour with oxygenated water and to eat nothing but liquid food.

In case of fever after operation we order him:

| | | |
|---|---|---|
| Antipyrin, | .65 | centigram ; |
| Tincture of aconite, | .52 | centigram ; |
| Sulphate of morphine, | .010 | centigram ; |
| Distilled water, | 124. | centigrams. |

Every hour a spoonful.

*October 24.*—The teeth were slightly movable, and the general state excellent.

*October 26.*—General state normal; the teeth appeared more movable.

*November 6.*—Teeth had become more firm; the state of health was good.

From that day we saw the patient once a week, and each day changed the ligature. The consolidation of the teeth kept progressing.

*January 15, 1898.*—The teeth were notably consolidated and the impression taken.

*February 15.*—The consolidation of the teeth was perfect.

I am very happy to express my thanks to Dr. Otto E. Inglis, who was so willing to assist me in the execution of the operation.—*L'Odontologie,* Paris.

